# New chemical biopsy tool for spatially resolved profiling of human brain tissue in vivo

**DOI:** 10.1038/s41598-021-98973-y

**Published:** 2021-09-30

**Authors:** Joanna Bogusiewicz, Katarzyna Burlikowska, Kamil Łuczykowski, Karol Jaroch, Marcin Birski, Jacek Furtak, Marek Harat, Janusz Pawliszyn, Barbara Bojko

**Affiliations:** 1 Department of Pharmacodynamics and Molecular Pharmacology, Faculty of Pharmacy, Collegium Medicum in Bydgoszcz, Nicolaus Copernicus University in Torun, Bydgoszcz, Poland; 2 Department of Neurosurgery, 10th Military Research Hospital and Polyclinic, Bydgoszcz, Poland; 3 Department of Neurosurgery and Neurology, Faculty of Health Sciences, Collegium Medicum in Bydgoszcz, Nicolaus Copernicus University in Torun, Bydgoszcz, Poland; 4 Department of Chemistry, University of Waterloo, Waterloo, Canada

**Keywords:** Analytical chemistry, Molecular neuroscience

## Abstract

It is extremely challenging to perform chemical analyses of the brain, particularly in humans, due to the restricted access to this organ. Imaging techniques are the primary approach used in clinical practice, but they only provide limited information about brain chemistry. Solid-phase microextraction (SPME) has been presented recently as a chemical biopsy tool for the study of animal brains. The current work demonstrates for the first time the use of SPME for the spatially resolved sampling of the human brain in vivo. Specially designed multi-probe sampling device was used to simultaneously extract metabolites from the white and grey matter of patients undergoing brain tumor biopsies. Samples were collected by inserting the probes along the planned trajectory of the biopsy needle prior to the procedure, which was followed by metabolomic and lipidomic analyses. The results revealed that studied brain structures were predominantly composed of lipids, while the concentration and diversity of detected metabolites was higher in white than in grey matter. Although the small number of participants in this research precluded conclusions of a biological nature, the results highlight the advantages of the proposed SPME approach, as well as disadvantages that should be addressed in future studies.

## Introduction

Neuroimaging methods enable the analysis of anatomical structures and functional changes in the brain. These techniques are based on wide range of physicochemical phenomena, such as electrical activity (e.g., encephalography), X-ray measurements (e.g., computed tomography), and magnetic field and computer-generated radio waves (e.g., magnetic resonance). Although the further development of these methods (e.g., the injection of radiocontrast or radiotracers) has enabled greater temporal and spatial resolution and analysis of functional changes in the brain, more invasive techniques are required for detailed explorations of the chemical composition of brain tissue^1,2^. Histopathology is the gold standard for diagnosing and analyzing diseases in clinical analysis, especially in neuro-oncology, followed by genetic and biochemical testing^3^. However, the main disadvantage of “out of organism” examination is the fact that enzyme activity does not automatically stop when the sample is removed^4^. To overcome this problem, some in vivo approaches, such as microdialysis (MD) and methods based on electrochemical detection, have been applied in neuroscience applications^5^.

The development of mass spectrometry (MS) has yielded new sampling techniques that can be applied for in vivo brain analysis. One such technique is rapid evaporative ionisation mass spectrometry (REIMS), which measures analytes present in “smoke” generated during the electrosurgical cutting of tissue. Another MS-based technique is probe electrospray ionization (PESI), which entails depositing a drop of water mixed with ethanol on the tissue surface. Analytes are extracted via this solution, and subsequently transferred to the electrospray ionization source of the mass spectrometer with a metal needle, which enables the ionization of the analytes when connected to high voltage^6,7^.

In recent years, solid-phase microextraction (SPME) has been successfully applied in in vivo studies^8^, including brain analyses of macaques^9^ and rats^10–13^. SPME is based on the interaction between compounds in a sample and an extractive sorbent, with extraction taking place until equilibrium is reached between the respective analyte concentrations in the sample and sorbent. Under certain conditions (i.e., when the volume of the sample is much higher than the volume of the extraction phase) the amount of analyte extracted is independent of the sample volume^14^. Therefore, SPME can be used successfully in in vivo studies where the volume of the sampled tissue or body fluid is not known or difficult to measure. The sampling procedure involves inserting a thin fiber coated with a biocompatible sorbent into the tissue for a predetermined period of time. Following sampling, the fiber is removed and subjected to a desorption step; after this desorption step has been completed, the extracted substances can be analysed. In vivo SPME is commonly referred to as a “chemical biopsy,” as there is no need to remove tissue or draw blood in order to analyze small metabolites^9,10,12,13,15^.

The present study aims to assess SPME's suitability for the spatially resolved metabolic characterization of living human brain tissue using a single sampling device and a short extraction time. Ultimately, the proposed strategy is intended to be applied for the simultaneous extraction of biomarkers from neoplasma and non-neoplastic tissue, as well as from injured and non-injured tissue. To simplify the experiment and uniform sampling procedure in this proof of concept study, a profiling of white and grey matter was selected.

## Materials and methods

### Chemicals

External calibrant Pierce LTQ Velos ESI Positive Ion Calibration Solution and Pierce LTQ Velos ESI Negative Ion Calibration was purchased from Thermo Scientific, while all other chemicals were purchased from Sigma Aldrich (Poznan, Poland). This research used LC–MS-grade isopropanol, methanol, water, acetonitrile, and mobile phase additives (formic acid, acetic acid, and ammonium acetate), and HPLC-grade chloroform. The probes were prepared using N,N-dimethylformamide ACS reagent and polyacrylonitrile.

### Materials

Three types of SPME fibers were used in the experiments: fibers coated with mix-mode ((MM) mixture of strong cation-exchange (SCX) and octyl (C8) particles) and octadecyl (C18) sorbents, both of which were kindly provided by Supelco, Merck; and hydrophilic-lipophylic balanced (HLB) fibers, which were manufactured in-house according to the protocol described in^16^. HLB particles were kindly provided by Waters (Wilmslow, U.K.).

### Human biological material

**Figure 1:**
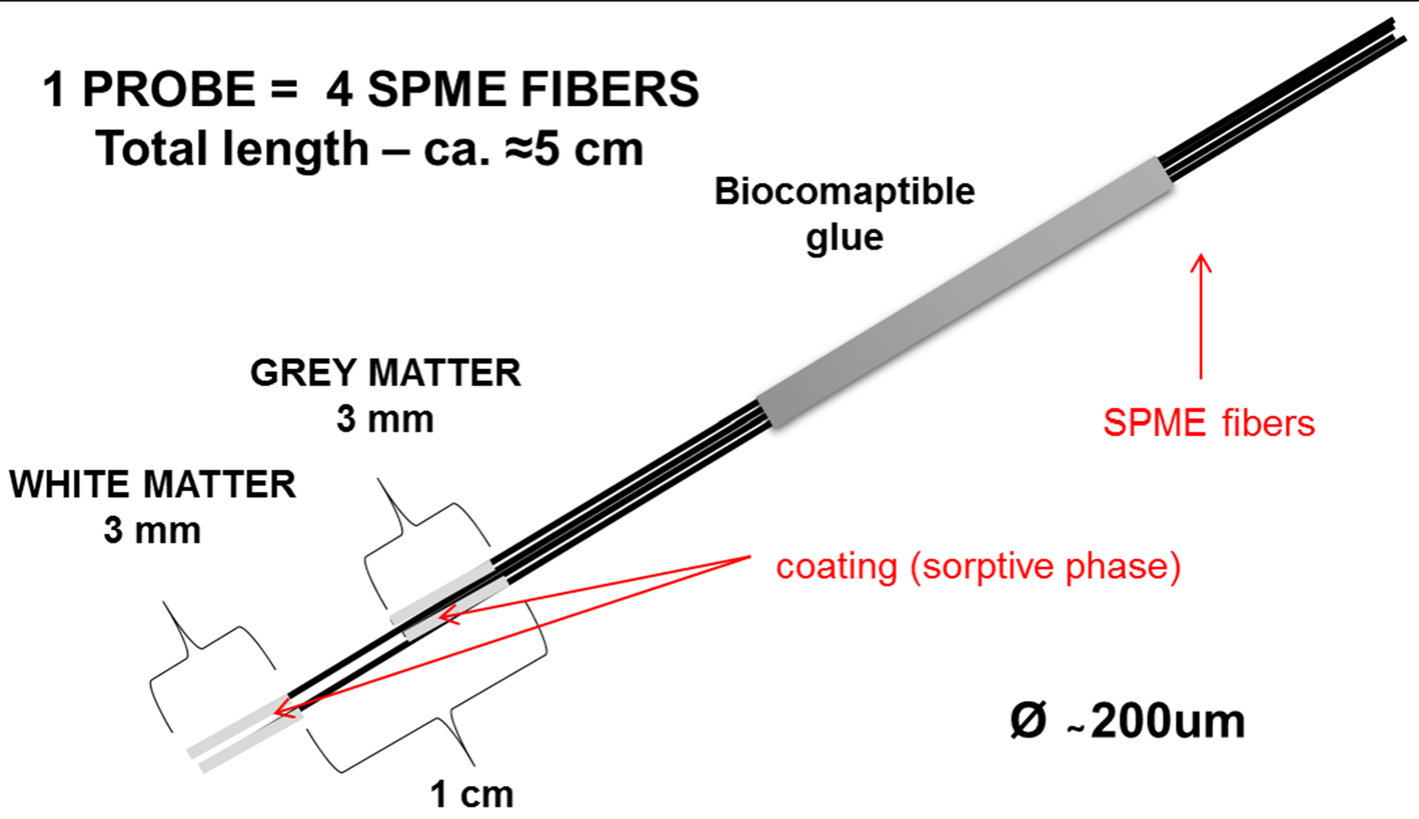
The construction of chemical biopsy probe.

Sampling was conducted at the 10th Military Research Hospital and Polyclinic in Bydgoszcz during brain biopsy procedures on patients with brain lesions localized at least 2 cm below the brain surface. Chemical biopsies (SPME samplings) were performed simultaneously on white and grey matter not affected by neoplastic changes using a specially designed device (Fig. 1). An acquisition time of 4 min was employed for sampling. Samples were obtained from eight patients whose detailed characterization is presented in Table S.A.1 of Supplementary Materials A. Written consent was obtained from each patient prior to undergoing the biopsy procedure.

In order to avoid additional brain damage, the SPME fibers were inserted along the planned trajectory of the biopsy needle. Sampling was performed by the neurosurgeon conducting the brain tumor biopsy procedure.

The study was approved by the Bioethical Committee of Nicolaus Copernicus University at Collegium Medicum in Bydgoszcz (KB 142/2017). The study protocol was performed in accordance with the relevant guidelines. The written informed consent and consent for publication was obtained from human participants.

### Solid-phase microextraction

#### General protocol for SPME

Fibers with a coating length of 3 mm were used for the experiments. To increase the coverage, four fibers were immobilized together at a set position, with polyacrylonitrile (PAN) being used as a biocompatible glue. Two longer fibers were introduced into the white matter of the brain, while the other two (1 cm shorter) were inserted into the grey matter. The construction of the device is illustrated in Fig. 1. Depending on the analysis, two types of sorbent were used: MM for metabolomics, and C18 for lipidomics.

#### Metabolomic analysis

**Figure 2:**
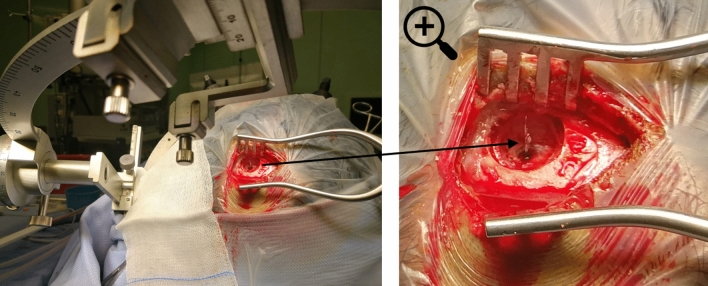
Chemical biopsy of brain tissue with the use of SPME probe.

Prior to sampling, the MM-fiber-based devices were preconditioned overnight in methanol:water (1:1 v/v) solution. After preconditioning, the fibers were transferred to individual glass vials, where they were sterilized in ethylene oxide in accordance with hospital protocols. The prepared devices were then used to conduct sampling during the biopsy procedure (Fig. 2). The sampling procedure (extraction of analytes) had a duration of 4 min. After extraction, the SPME device was removed and washed for 3 s. via static immersion in water. Following washing, the device was dismounted, and the fibers were separated, securely placed in labeled vials, and stored at − 30 °C until liquid chromatography-high resolution mass spectrometry (LC-HRMS) analysis. Immediately prior to instrumental analysis, the metabolites were desorbed from the fiber using an acetonitrile:water (80:20 v/v) solution for 1 h with agitation at 1200 rpm. To increase recovery rates, two fibers used to the sample the same structure were desorbed together in one vial with 150 μl of desorption solvent.

#### Lipidomic analysis

Before being used, the C18-fiber-based devices were immersed in a chloroform:methanol (2:1 v/v) solution for 45 min with agitation at 1200 rpm, and then preconditioned overnight in methanol:water (1:1 v/v) solution. Following preconditioning, the fibers were sterilized using the same protocol as was used for the MM devices. Similarly, the experiments using the C18-fiber-based devices also utilized the same sampling and storage procedures used in the experiments for the MM-based devices. Directly before instrumental analysis, the analytes were desorbed from the fibers via submersion in 150 μl of an isopropanol:methanol (1:1 v/v) solution for 1 h, with agitation at 1200 rpm. As in the metabolomics analyses, the two fibers used to sample each structure (i.e., two fibers for grey matter, and two for white matter) were desorbed together in one vial in order to increase recovery. Silanized glassware was used for the lipidomic analyses.

#### Selection of SPME coating for lipidomics

The effectiveness of the sorbents used in the lipidomic studies was assessed using brain tissue from a C57BL/6 mouse. According to European Union regulations, permission from the Local Ethical Commission is not required for the *post mortem* use of animal tissue or organs for scientific purposes. The mouse was euthanized via cervical dislocation, and its brain was immediately collected and stored at − 80 °C until analysis. Prior to sampling, the brain was thawed at room temperature, and then SPME probes were inserted into the hypothalamus for 15 min. The six fibers used for sampling were preconditioned overnight in a solution consisting of methanol:water (1:1 v/v) (three C18 and three HLB). After sampling, the fibers were desorbed under the same conditions used in the lipidomic analyses (described above).

### Liquid chromatography-high resolution mass spectrometry (LC-HRMS)

Liquid chromatography coupled with high-resolution mass spectrometry (Q Exactive Focus) was performed. Data acquisition was performed using dedicated Thermo Scientific software, namely, Xcalibur 4.2 and Free Style 1.4. The mass spectrometer was externally calibrated, resulting in mass accuracy of < 2 ppm.

Samples were randomized within the sequence, and instrument performance was monitored by running pooled quality control (QC) samples composed of 10 µl of each sample every 4–5 injections. Instrumental blanks (desorption solvent) and extraction blanks (negative control) were also injected every 4–5 samples.

The metabolomic analyses utilized reversed-phase liquid chromatography (RPLC) and hydrophilic-interaction liquid chromatography (HILIC) in positive and negative ion mode. The protocol used in these analyses has been detailed in^17,18^.

The structure of the detected compounds was confirmed by matching their fragmentation patterns to spectra libraries at a mass accuracy of < 3 ppm. To this end, full MS/dd-MS2 discovery mode was used. The following fragmentation parameters were utilized for metabolomic analysis: mass resolution—35,000 FWHM; AGC target—2E4; minimum AGC—8E3; intensity threshold—auto; maximum IT—auto; isolation window—3.0 m/z; stepped collision energy—10%, 20%, and 40%; loop count—2; and dynamic exclusion—auto.

Reversed-phase liquid chromatography (RPLC) and hydrophilic interaction chromatography (HILIC) in positive ion mode were conducted for the lipidomic analyses. The protocol used for these analyses is detailed in^17^ and ion source conditions were given in^18^.

As with the metabolomics analyses, the structure of the detected compounds was confirmed by matching their fragmentation patterns to spectra libraries at a mass accuracy of < 3 ppm. Full MS/dd-MS2 discovery mode was used for this purpose. The fragmentation parameters used for lipidomic analyses were as follows: mass resolution—35,000 FWHM; AGC target—2E4; minimum AGC—8E3; intensity threshold—auto; maximum IT—auto; isolation window—3.0 m/z; stepped collision energy—10%, 20%, and 40%; loop count—2; and dynamic exclusion—auto.

### Data processing and statistical analysis

Compound Discoverer 2.1. (Thermo Fisher Scientific, San Jose, California, USA) software was used for metabolomics data processing and statistical analysis. The mass tolerance window was set to 3 ppm, the peak intensity was set to > 10 000, the signal to noise threshold was set to 3, and the max sample-to-blank ratio was set at > 5. Data was subjected to autoscaling, and the QC-based area was used for correction (min 50% coverage, max 30% RSD in QC, normalization by constant mean, fold change > 2 and P < 0.05). After aligning the peaks, gap filling was applied to fill in any missing values by a very small peak at the level of spectrum noise for the compound.

Following the annotation step, the compounds were searched for biological characteristics against HMDB, PubMed, Chemspider and BioCyc databases. Next, a compound table, including all chromatography modes, was created and entered into Metaboanalyst online software, and a pathway search was performed using the Kyoto Encyclopedia of Genes and Genomes (KEGG) database^19^. Fragmentation spectra were confirmed via online databases such as LIPID MAPS, HMDB, METLIN, and mzCloud.

The data for the lipidomic studies was processed using LipdSearch 4.1.30 (Thermo Fisher Scientific, San Jose, California, USA) with the accuracy set to 3 ppm and the intensity threshold set to 10 000. With the exception of phosphatidic acids (PA), phosphatidylinositols (PI), and fatty acids (FA), which are more characteristic for negative ion mode not used in this study, all classes of lipids were included into the search range for RPLC-HRMS^20^. In contrast, for HILIC-HRMS only glycerolipids were excluded. The searched ion adducts consisted of H^+^, NH_4_^+^ and Na^+^. An m-score of 10 and a retention time tolerance 0.25 min were utilized as the alignment settings. The following parameters were used to filter obtained results: for extraction control, an area coefficient of variation (CV) of < 30 and not equal to 0; a QC and extraction blank area ratio > 20; and a peak quality of > 0.85 for at least one of the studied groups.

To filter false positive lipid ID from LipidSearch results we applied identification grade filtering. Only lipids of which fatty acid chains and class were identified completely and lipids of which class and some fatty acid chains were identified (grade A and B, respectively).

Multivariate statistical analysis was performed using the online Metaboanalyst software package^21^. Missing values were estimated and replaced by small numbers (i.e., half of the minimum positive values in the study data). Logarithmic transformation and autoscaling were also applied. Finally, the following multivariative approaches were utilized: principal component analysis (PCA), partial least squares discriminant analysis (PLS-DA), and volcano-plot.

Basic statistical analysis was performed using Statistica 13.3 PL (StatSoft, Inc., Tulsa, Oklahoma, USA) software, while homogeneity was assessed via Levene’s test and normality was checked using the Shapiro–Wilk test. The Student's t-test was used to evaluate the statistical significance of the differences between the studied groups, and the Mann–Whitney U test was employed when hypotheses about the normality and homogeneity of variance were not fulfilled. A p value of 0.05 was set as a threshold of statistical significance, and an F-test was used to compare the standard deviations.

Peaks area for lipidomic data were normalized on the total peak area for all lipids and these values were used for detailed statistical analysis.

## Results

### Chemical biopsy device

The four-fiber SPME device was successfully used to perform chemical biopsies in the examined patients (Figs. 1, 2). The SPME device, which is smaller than the biopsy needle, was inserted prior to the standard biopsy procedure along the trajectory planned for the biopsy needle in order to prevent any excessive tissue damage. The entire SPME procedure took less than 5 min. It is also worth noting that no mechanical damage (e.g., crumbling of the sorbent, fiber fractures, decomposition) to the device was observed.

### Metabolomics

The first untargeted analysis was performed using the metabolomics protocol. A total of 75,449 features were detected after running all four LC-HRMS modes. After filtering out the background noise and the unstable features, a total of 2071 compounds were identified in the analyzed samples, which constituted 541 hits within the ChemSpider database (Figs. SA1-SA4 of Supplementary Materials A). According to Metabolomics Standards Initiative (MSI) scale, the mentioned compounds were classified on the 4th level. A comparison of the number of compounds detected in the grey and white matter samples revealed no significant differences, with an average grey-to-white compound ratio of 0.931. Greater variations in analyte coverage were usually detected inter-individually for the given matter rather than between the two types of brain matter in subsequent individuals (Fig. SA5 of Supplementary Materials A).

Further fragmentation of detected analytes which enabled to attain 2nd level of confidence according to MSI, revealed that the majority of confirmed compounds were lipids (Tables SA2 and SA3 of Supplemenrary Materials A).

### Lipidomics

#### Selection of SPME coating for lipidomics

The first step in the lipidomic analysis process was to determine the optimal sorbent type, as this would ensure maximum effectiveness. To this end, fibers coated with C18 and HLB particles were used to obtain samples from the mouse brain specimens. The fibers were then subjected to HILIC-HRMS analysis, which detected 164 lipids, and RPLC-HRMS analysis, which detected 463 (Table SB1 and SB2 of Supplementary Materials B, respectively). Only 12.8% of the normalized peak areas for the compounds detected via HILIC-HRMS, and 6.5% of the compounds detected via RPLC-HRMS analysis were statistically different between the two types of sorbent (Table SB1 and SB2 of Supplementary Materials B). The average normalized area under the peak for almost all of the significantly differing compounds was larger when sampling was performed using the C18 fibers. Additionally, an F-test was conducted in order to compare the two coatings’ coefficients of variation (CV) for the extracted compounds. The results revealed similar CVs for the majority of detected metabolites; however, sampling with the C18 fibers yielded statistically significantly higher CVs for 7.4% of the features in HILIC-HRMS and 16.4% of the features in RPLC-HRMS. When the HLB fibers were used, CVs were only higher for 2.8% of the detected putative analytes in reversed-phase chromatography (Table SB1 and SB2 of Supplementary Materials B). Ultimately, the C18-coated fibers were selected for use in the human subject experiments, as they provided larger average areas under the peaks.

#### Human brain analysis

A wide range of putative lipid species (phospholipids, glycerolipids, and sphingolipids) were detected in positive ion mode for HILIC-HRMS and RPLC-HRMS chromatography (189 and 245 compounds, respectively). RPLC-HRMS analysis mainly enabled the detection of triglycerides (TG) (Table SA4 of Supplementary Materials A), while HILIC-HRMS chromatography facilitated the analysis of more hydrophilic species, mainly phospholipids (Table SA5 of Supplementary Materials A). Multivariate analysis conducted with the online Metaboanalyst software package revealed weak separation between the white and grey matter (Figs. SA6 of Supplementary Materials A). As with the metabolomics results, PCA did not show any separation between the two types of matter. In addition, the PCA findings showed that the data points representing grey matter were closer to each other than the ones for white matter.

**Figure 3:**
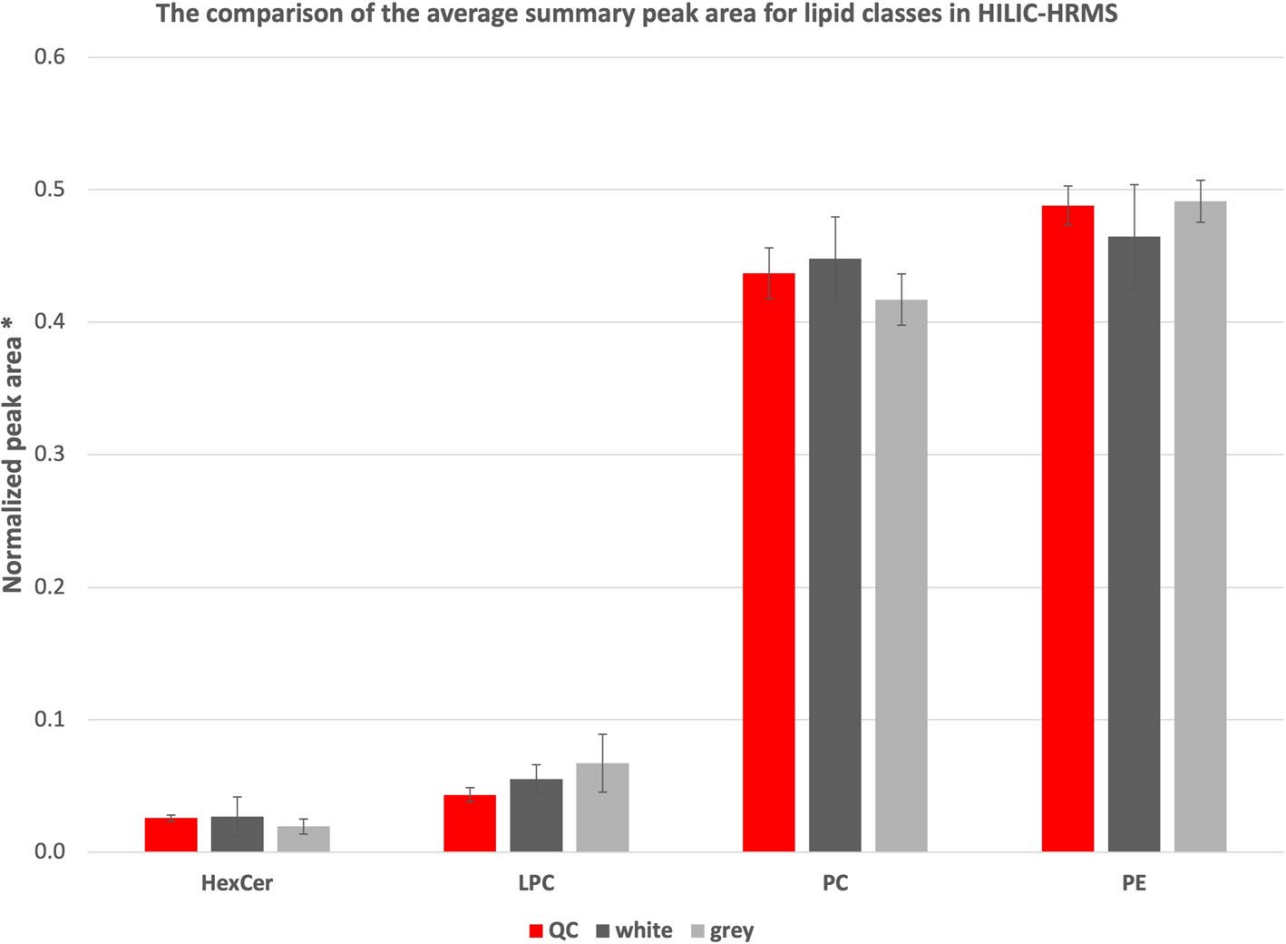
The comparison of the average summary peak area for lipid classes in HILIC-HRMS. Plots with mean normalized peak areas for lipid classes with with whiskers representing standard deviation. *Ratio of the peak area and total peak area for lipids. *HexCer*—Hexosyl ceramide, *LPC*—Lysophosphatydylocholines, *PC*—Phosphatydylocholines, *PE*—Phosphatydyloetanoloamines, *QC*—Quality Control.

**Figure 4:**
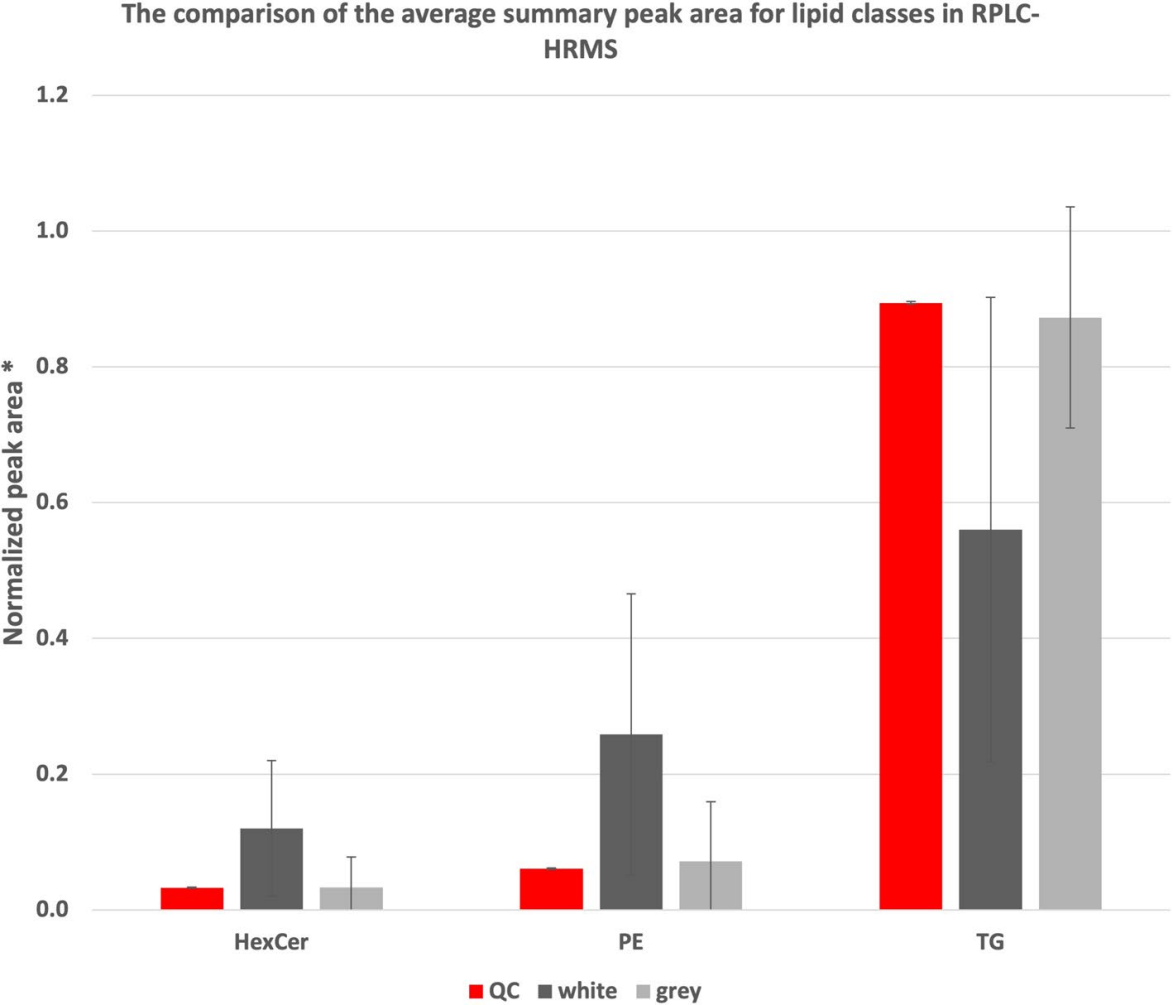
The comparison of the average summary peak area for lipid classes in RPLC-HRMS. Plots with mean normalized peak areas for lipid classes with with whiskers representing standard deviation. *Ratio of the peak area and total peak area for lipids. *HexCer*—Hexosyl ceramide, *PE*—Phosphatydyloetanoloamines, *TG*—Tryglicerides, *QC*—Quality Control.

Next, statistical analysis was conducted on the compounds with confirmed structures. Data obtained with HILIC chromatography did not revealed significantly different levels of phospholipids and hexoyl ceramides (HexCer) in studied structures (Fig. 3). In more in-depth terms, three phosphatydylocholines (PC) and two phosphatydyloetanoloamines (PE) were more abundant in grey matter with p < 0.05 (Table SA5 of Supplementary Materials A). Interestingly, the amount of detected triglycerides in the grey matter was over two times higher than in the white matter (Fig. 4). More in-depth analysis of individual lipid species can be found in Table SA4 of Supplementary Materials A. As can be seen, majority of TGs were present in grey matter at slightly higher levels as in white matter. Similarly, all the phospholipids analyzed with RPLC-HRMS were present in white matter at concentrations at least three times as high as in grey matter. HexCer was also more abundant, occurring about 2 times as often in white matter compared to grey matter.

## Discussion

The introduction of new mass-spectrometry-based techniques to neuroscience has created a wide array of new opportunities within this discipline^22,23^. Nonetheless, many challenges related to areas such as safety, biocompatibility, and practicability must still be overcome. The present article explores chemical biopsy as a new tool that can be used in neurochemistry applications. Previous ex vivo and in vivo investigations of animal brains have demonstrated that some of this technology’s unique features may be able to fill the gaps present in existing methods^10,11^. In addition, prior findings have shown that chemical biopsy is safe for use in laboratory animal studies, and enables the non-depletive analysis of labile endogenous substances, as well as metabolism quenching due to its ability to restrict the access of enzymes to absorbed substances^10,13,15,24^.

This paper documents the first application of chemical biopsy for the analysis of human brain tissue in vivo*,* which was achieved by introducing a few adjustments to previously reported protocols. Firstly, it was necessary to sterilize the SPME fibers before inserting them into the human participants’ brains. To this end, ethylene oxide was used in this study, but other research has shown that steam sterilization can be used as well^25^.

Another modification to previous SPME protocols made in this research was the compromise that was struck between analytical needs and practicality in a clinical setting. Specifically, the selected sampling time must be long enough to ensure adequate sensitivity and data quality, but short enough to be acceptable for the neurosurgeon and the patient. The length of the extractive phase coating on the probe must also be sufficient to attain reasonable recovery of the metabolites, but not too long to fit within the selected location. A literature review revealed that a sampling time of 15–30 min is usually used in metabolomics studies^26^. However, since the extractions were performed on conscious patients, prior to the actual biopsy procedure, the extraction time used in this research could not be longer than a few minutes in duration. Moreover, equilibrium time is established more quickly in complex matrices than in simple solutions (like phosphate buffered saline (PBS) or agarose gel) due to the fact that analytes are bound by tissue components (e.g., proteins), which in turn act as a local reservoir for the molecules to be extracted^27^. Based on these findings, it is easy to conclude that even a short extraction time (e.g., 5 min) might be sufficient to achieve satisfactory recovery when extraction is performed from a complex matrix^27^.

Unlike SPME, gold standard method in neuroscience—MD requires a relatively long sampling time, as it takes over 30 min to collect a sufficient amount of dialysate to enable the determination of target compounds. Furthermore, a special cannula must be installed in the patient’s skull 3–4 days prior to the experiment in order to allow the MD probe to be introduced to the brain. Attempts to modify this technology led to the development of push–pull probes (PPP), which enable faster analysis, but lower sensitivity and accuracy^28^. Compared to MD and SPME, PPPs and electrochemical methods guarantee better temporal resolution, although push–pull probes are still prone to capillary clogging^28,29^. Additionally, while electrochemical methods are able to return results in a few seconds, they only enable the analysis of selected substances, which may considerably reduce the scope of research^28,30^.

As previously mentioned, extraction time and coating length must be customized in order to perform extractions from brain structures using SPME. The grey matter is a thin layer on the surface of the brain ranging between 1.0 and 4.5 mm in thickness (approximately 2.5 mm) depending on the region^31^. In the present study, probes with a coating length of 3 mm were selected in order to enable the extraction of metabolites from grey matter with maximum precision and acceptable sensitivity. However, to further increase sensitivity without compromising extraction time, a modification was introduced to the design of the sampling device. Since it is possible to increase analyte extraction by increasing the surface area of the extraction phase (coating)^14^, recovery was enhanced by sampling each brain structure with two fibers simultaneously (Figs. 1, 2) following by desorption of both fibers together in one vial. Another option is the change of the device’s geometry to a thin-film format^24^ but such devices are too invasive for use in brain studies. Nonetheless, it is important to note that the range of extracted metabolites was still limited, and, consistent with the previous research, for instance neurotransmitters were not observed in untargeted analyses^10,11^. However, it is possible to extract neurotransmitters with SPME by optimizing the analytical protocol for that specific group of substances^10,15^.

Some approaches using SPME fibers were employed so far to obtain spatial resolution results. One of them was the assessment of pollutants in fish tissue samples with segmented SPME fibers to simultaneously sample adipose and muscle tissues^32^. Recently, another approach to spatial resolution analysis was presented by Lendor et al., who analyzed the fluoxetine profile in rat brains using SPME fibers for extraction, and desorption electrospray ionization (DESI) coupled to mass spectrometry for target analyte detection along the probe^33^.

The analytical methods most commonly used in in vivo neurochemical studies generally do not provide spatial resolution of brain structures that is as detailed as the SPME-DESI approach^33^. With MD probes-even those with a diameter similar to SPME fibers (about 200 μm)—it is impossible to obtain comparable results due to the method’s different governing principles. Thus, sampling must be carried out in a different way, which usually results in slower spatial resolution or time delay (i.e., sampling the grey and white matter sequentially rather than simultaneously)^28,34^. Moreover, if sampling from two locations is planned, two cannulas outfitted with MD probes must be used, which will increase the invasiveness of the procedure and impact spatial resolution. Additionally, the MD probe needs to be connected to the device that pumps the perfusate during the sampling procedure. This means that a two single-channel-syringe pumps or one two-channel-syringe pump would be required in order to analyze two brain locations, which creates a need for additional space in the surgery room to accommodate the necessary extra equipment.

Distinct differences in the metabolomic or lipidomic profiles of the two studied brain matters were expected, but PCA did not show clear separation (Fig. SA5, Fig. SA6 of Supplementary Materials A). The patients from whom samples were collected in this study constituted a heterogeneous group in terms of age, sex, type, and location of brain lesion (Table SA1 of Supplementary Materials A). This could affect the repeatability of the obtained results and contribute to the relatively high variation observed in the average peak areas (Table SA2-SA5 of Supplementary Materials A). For these reasons, biochemical in vivo brain studies are almost exclusively performed on laboratory animals, wherein entire neurosurgical procedures are designed for the research at hand. The current study was conducted on a small cohort, which makes it difficult to draw solid conclusions. Indeed, our results only verify the concept and identify drawbacks that should be addressed in future studies seeking to analyze a larger number of patients.

Mainly lipids were identified in the metabolomic samples (Table SA2., and Table SA3 of Supplementary Materials A) which is in line with previous reports identifying lipids species as the predominant component in brain tissue composition^6,15,31,35^. With regards to cerebral lipids, it is worth mentioning that MRI, a clinically approved method of differentiation of brain structures and detecting lesions, is mainly based on differences in lipid content (cholesterol and phospholipids)^36^. Prior findings have also shown that white matter contains about 60% more lipids and 10–15% less proteins than grey matter^36^. Similar observations regarding the prominent role of lipids in brain cells have been made using other mass-spectrometry-based methods, including REIMS and DESI^22,37–39^. SPME on the other hand was reported as a complementary technology to MD, as it covers a broader hydrophobic range of metabolites (e.g., lipids), while MD is suitable for highly polar species (e.g., amino acids)^10,11^. Both methods were compared by the performance of untargeted metabolomic profiling as well as targeted analysis of neurotransmitters obtained from brain of living rats.

Solid-phase microextraction makes it possible to tailor the protocol to the metabolites of interest, as it allows researchers to employ more selective coating and desorption parameters, and it can be coupled with appropriately optimized analytical instrumentation. In the present study, the results of the metabolomic analysis indicated a need for more in-depth lipidomic analysis. In order to better assess the lipid composition of the examined tissues, two types of sorbent were tested. First, fibers coated with C18 were analyzed, as they have been used in lipidomic studies previously, and they have a high affinity towards hydrophobic species^12,40,41^. The second type of fiber that was selected for analysis was HLB-sorbent-coated fibers, as previous studies have demonstrated their high level of performance^41,42^. The results of our comparison showed that the peak areas of detected features were larger in the samples collected with the C18 fibers, which indicated that this sorbent offered slightly better recoveries. The areas of only a few compounds showed a statistical significant difference (Table SB1 and Table SB2 of Supplementary Materials B). Based on this finding, the lipidomic analysis was carried out using the C18 fiber. This is consistent with the previous work which presented the use of the C18 sorbent for lipidomic analysis, and a mixed-mode extractive phase for metabolomic analysis^11^.

Despite the lack of statistically significant separation between the two types of brain matter determined by PCA (Fig.SA6 of Supplementary Materials A), the results nevertheless showed the characteristic patterns of lipids in those structures. For instance, the results showed that phospholipids were present in greater level in the white matter while TGs were elevated in grey matter (Figs. 3, 4 and  Table SA4 and Table SA5 of Supplementary Materials A). Nevertheless, due to small cohort of patients and high diversity of samples, detailed discussion on biological role and implications to spatial distribution were not included in the current work.

The research described herein demonstrates that it is possible to achieve spatially resolved chemical characterization of a living human brain in a fast and low invasive manner, thus not disturbing other medical procedures (e.g., biopsy). The sampling device can be customized into a personalized diagnostic tool, and the proposed protocol can be applied in numerous clinical settings when supported by imaging data. In addition, this research proves that the proposed method complements those already used in neuroscience, for example, microdialysis, push–pull analysis, and electrochemical methods. Furthermore, the findings of this research also show that chemical biopsies performed with SPME probes enable more in-depth chemical analysis of brain tissue compared to other clinically available methods (e.g., MRI, biopsy). Not only is SPME capable of extracting lipids and more polar metabolites, but its lipid-characterization capabilities are also unique compared to other in vivo sampling devices. The results presented in this paper indicate that the proposed tool could be potentially used as a medical device, as it causes minimum damage and features operational parameters that are compatible with surgical procedures.

Finally, it should be emphasized that more portable reading devices-rather than mass spectrometers using optical spectroscopic techniques, such as fluorescence or Raman could be used in targeted determinations in order to create a technology that is more compatible with bedside or operational theatre applications^43^.

## Supplementary Information

Supplementary Information 1.

Supplementary Information 2.
