# Supplementary material for: New chemical biopsy tool for spatially resolved profiling of human brain tissue in vivo
Source: Sci Rep. 2021 Sep 30;11:19522. doi: 10.1038/s41598-021-98973-y (PMC8484280; doi:10.1038/s41598-021-98973-y)
Supplement: Supplementary file 1 — Supplementary Information 1. [file 41598_2021_98973_MOESM1_ESM.pdf]

New Chemical Biopsy Tool for Spatially Resolved Profiling of Human Brain Tissue in vivo

Joanna Bogusiewicz[1], Katarzyna Burlikowska[1], Kamil Łuczykowski[1], Karol Jaroch[1], Marcin Birski[2], Jacek Furtak[2], Marek Harat [2,3#], Janusz Pawliszyn[4#], Barbara Bojko[1#]

1. Department of Pharmacodynamics and Molecular Pharmacology, Faculty of Pharmacy, Collegium Medicum in Bydgoszcz, Nicolaus Copernicus University in Torun, Jurasza 2 Street, 85-089 Bydgoszcz, Poland
2. Department of Neurosurgery, 10th Military Research Hospital and Polyclinic, Powstancow Warszawy 5 Street, 85-681 Bydgoszcz, Poland
3. Department of Neurosurgery and Neurology, Faculty of Health Sciences, Collegium Medicum in Bydgoszcz, Nicolaus Copernicus University in Torun, Ujejskiego 75 Street, 85-168 Bydgoszcz, Poland
4. Department of Chemistry, University of Waterloo, 200 University Avenue West, Waterloo, Ontario N2L 3G1, Canada

#corresponding authors

Marek Harat – e-mail: harat@10wsk.mil.pl; phone: +48261417094; Janusz Pawliszyn – e-mail: janusz@uwaterloo.ca; Barbara Bojko – e-mail: bbojko@cm.umk.pl; phone: +48525853564

Figures

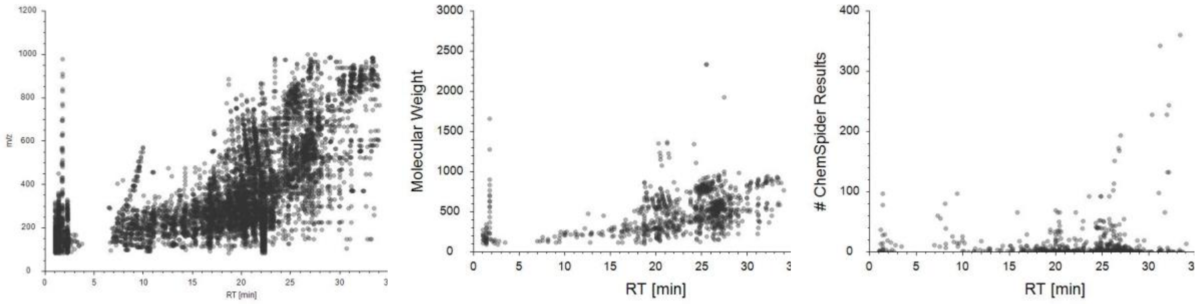

Fig. SA1. Scatter plots representing A. number of features, B. compounds and C. Chemspider database hits acquired in PFP (+) metabolomics analysis.

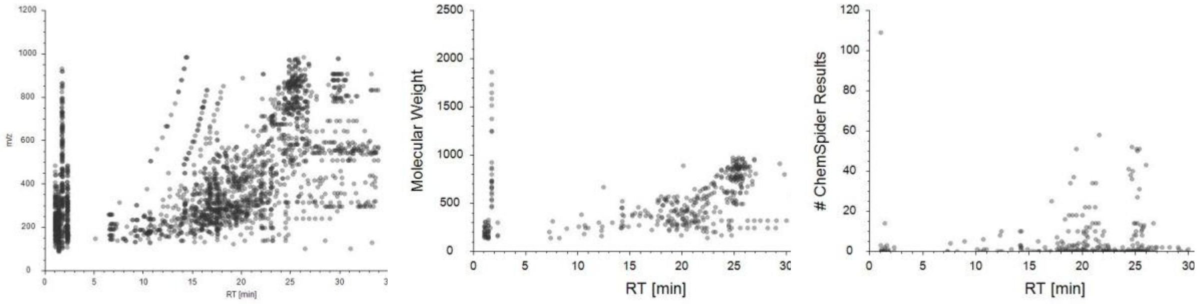

Fig. SA2. Scatter plots representing A. number of features, B. compounds and C. Chemspider database hits acquired in PFP (-) metabolomics analysis.

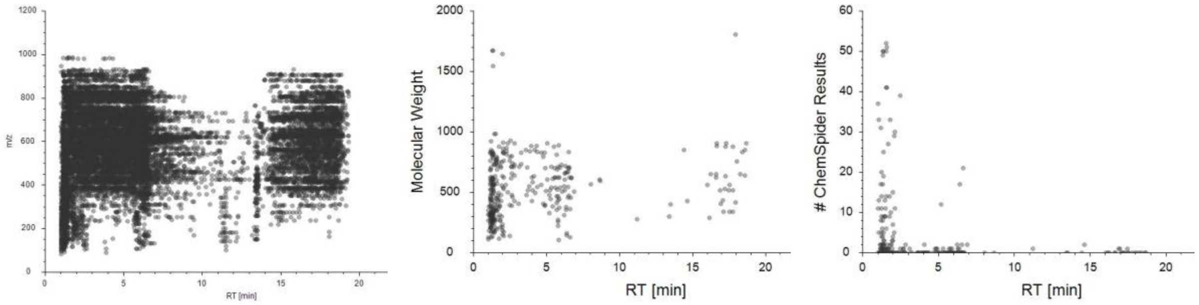

Fig. SA3. Scatter plots representing A. number of features, B. compounds and C. Chemspider database hits acquired in HILIC (+) metabolomics analysis.

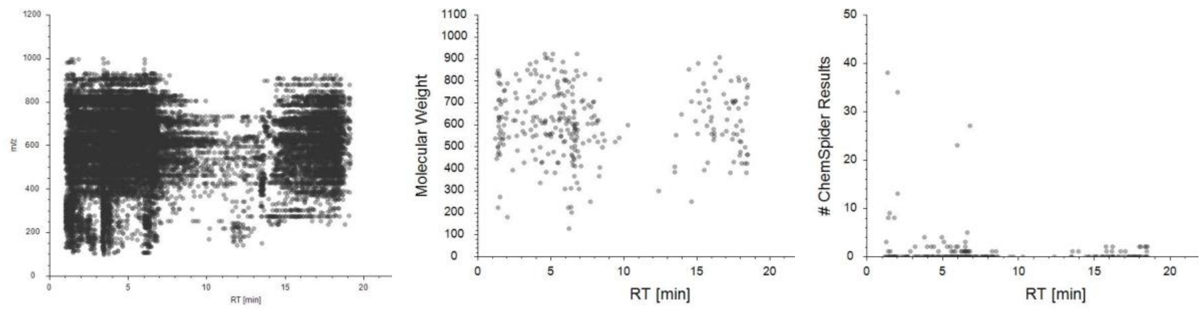

Fig. SA4. Scatter plots representing A. number of features, B. compounds and C. Chemspider database hits acquired in HILIC (-) metabolomics analysis.

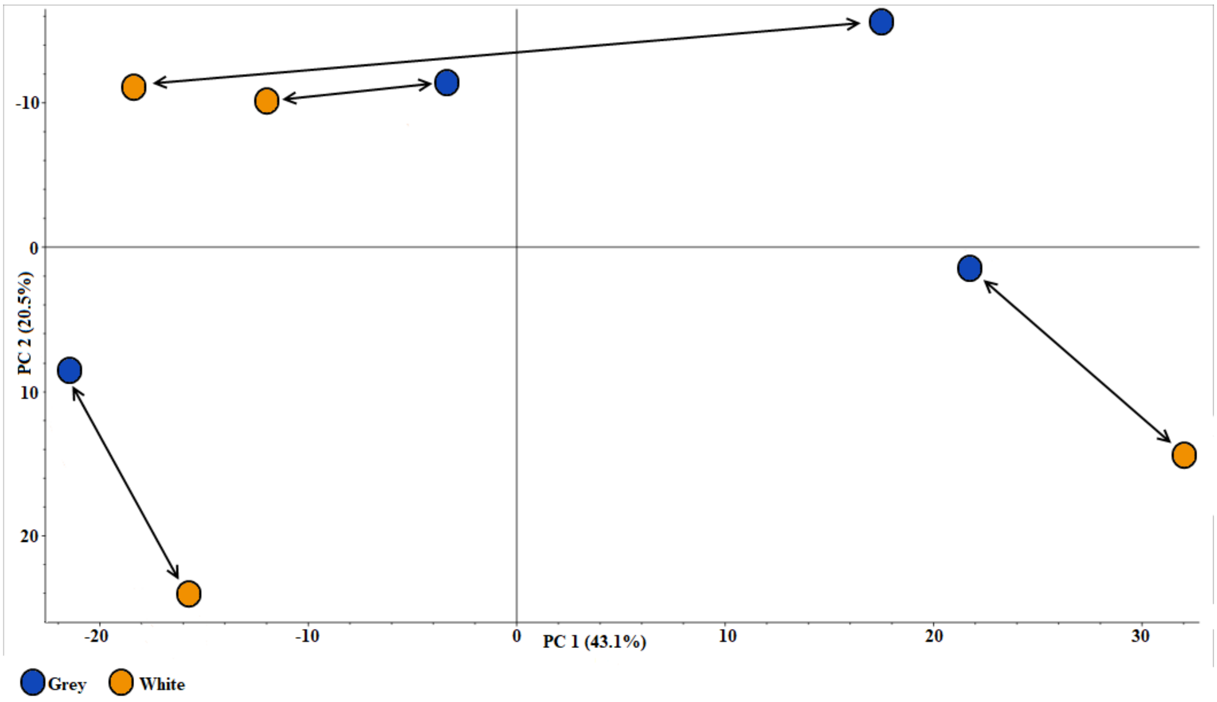

Fig. SA5 The Principal Component Analysis of white (blue) and grey (orange) matters for metabolomics data obtained with use reversed-phase liquid chromatography (RPLC) high resolution mass spectrometry (HRMS). Arrows represents samples from the same individual.

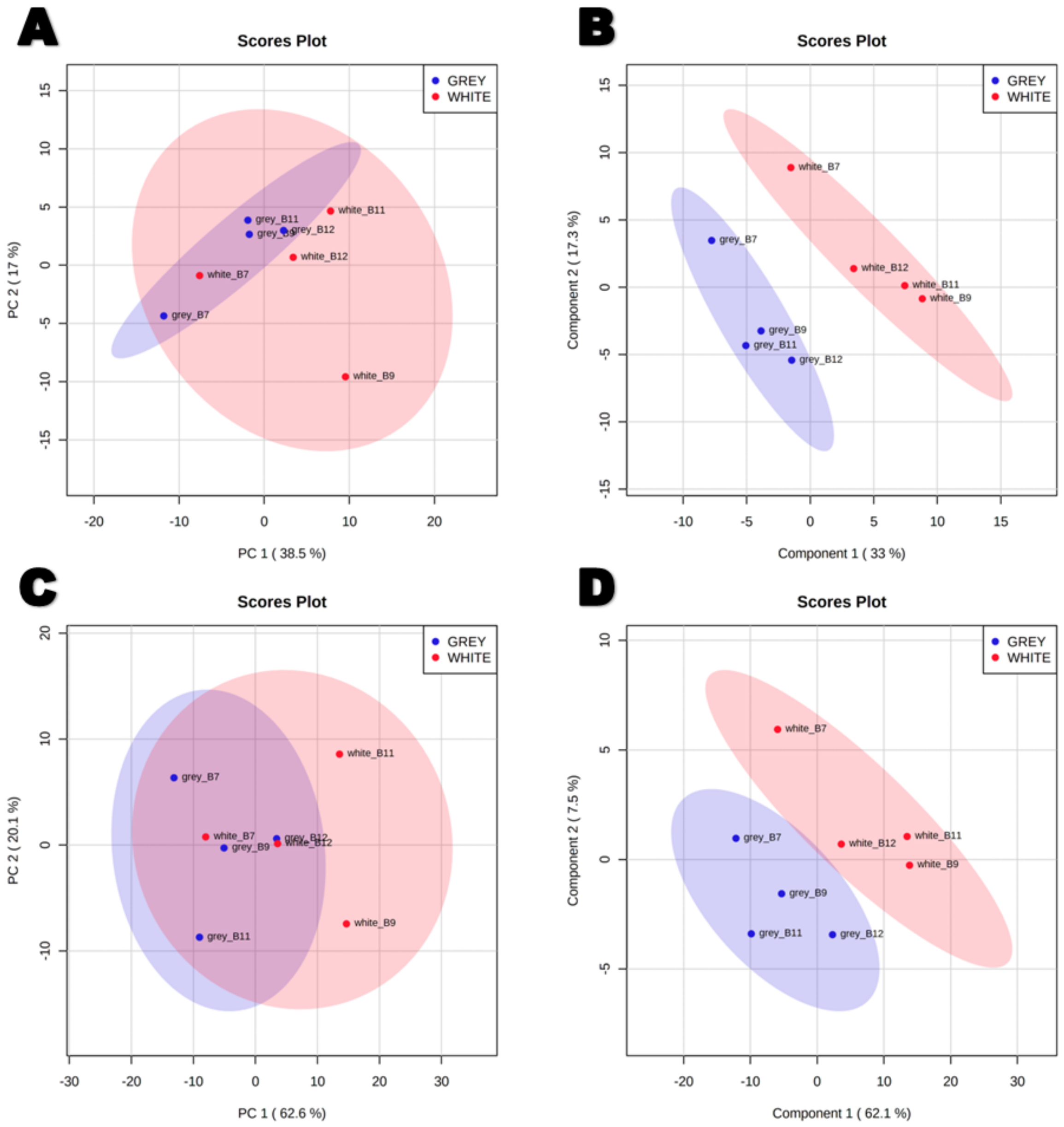

Fig. SA6. Chemometric analysis of data obtained with use of hydrophilic-interaction liquid chromatography high resolution mass spectrometry (HILIC-HRMS) for lipidomic data.

A. principal component analysis (PCA); B. partial least squares discriminant analysis (PLS-DA), plot parameters: 1 component: R2: 0.659; Q2: -0.072; 2 component: R2: 0.957; Q2: -0.129; and reversed-phase liquid chromatography high resolution mass spectrometry (RPLC-HRMS) for lipidomic data C. PCA; D. PLS-DA, plot parameters: 1 component: R2: 0.447; Q2: 0.060; 2 component: R2: 0.881; Q2: 0.049.

The parameters of PLS-DA for HILIC-HRMS showed over-fitting of the model, while separation was slightly better for the RPLC-HRMS analysis. It was observed that the samples taken from patient B12 were located very close to each other (almost overlapping), which may lead to the assumption that, in this case, sampling was performed only from one type of matter.

Tables

Table SA1 Patient and tumor sampling characteristics.

Sample ID	Histopathologic diagnoses	Brain tumor location	Age	Sex	Sorbent	Type of Analysis
B2	Diffuse large B-cell lymphoma	Corpus callosum	55	female	MM	metabolomics
B3	High- grade glioma (WHO grade III)	Left parietal lobe	46	female	MM	metabolomics
B4	Metastatic breast adenocarcinoma	Left temporal lobe	57	female	MM	metabolomics
B5	Glioblastoma, IDH- wildtype	Deep structures of the right hemisphere	66	male	MM	metabolomics
B7	Glioblastoma, NOS	Right frontal lobe	58	male	C18	lipidomics
B9	Gemistocytic astrocytoma, IDH- wildtype	multifocal tumor of the left hemisphere	55	male	C18	lipidomics
B11	hemorrhagic stroke (non-neoplastic cells)	Left thalamus	63	Female	C18	lipidomics
B12	Diffuse large B- cell lymphoma	Right fronto-parietal region	64	Female	C18	lipidomics

C18 – octadodecyl fibers; MM – mix mode fibers; NOS – not otherwise specified; WHO – World Health Organization

Table SA2 Metabolites detected in HILIC-HRMS negative ion mode analysis and confirmed with fragmentation pattern.

Name + adduct	Observed m/z	Retention Time [min]	QC		White		Grey		WHITE/ GREY
			Mean	CV [%]	Mean	CV [%]	Mean	CV [%]	
N-Nonanoylglycine -H	214.14453	12.3	1.93E+05	11.43	5.55E+04	65.71	8.71E+04	38.11	0.6
Tridecanoylglycine -H	270.20792	15.9	2.32E+05	8.22	1.22E+05	112.89	1.57E+05	45.58	0.8
16-Hydroxyhexadecanoic acid -H	271.22812	19.5	4.36E+04	5.05	2.24E+04	29.99	6.61E+04	66.94	0.3
Acetoxyacorenone -H	277.18127	18.4	1.42E+04	13.06	8.79E+03	72.27	1.00E+04	101.17	0.9
(Arabinosylamino) (glucosylamino)propanenitrile –H	378.15161	14.2	2.46E+04	3.36	1.38E+04	79.41	1.44E+04	40.03	1.0
LPE(18:1) -H	478.29468	18.2	1.80E+04	4.68	2.25E+04	79.79	2.20E+04	53.59	1.0
LPE(20:4) -H	500.27963	17.7	1.99E+04	8.69	1.95E+04	61.27	2.37E+04	47.96	0.8
PE(22:4) -H	528.31079	18.6	1.59E+04	3.62	2.02E+04	68.45	1.87E+04	51.18	1.1
PE(22:6) -H	542.27942	17.8	2.91E+04	3.08	1.96E+04	99.82	2.50E+04	27.37	0.8
PE(36:2) -H	742.54120	25.4	1.52E+05	3.05	2.06E+05	57.12	1.65E+05	59.86	1.3
PE(38:6p) -H	746.54514	24.7	2.26E+05	3.82	1.83E+05	95.83	1.91E+05	80.49	1.0
PE(38:5p) -H	748.53040	25.1	3.30E+05	3.82	3.67E+05	61.51	3.10E+05	39.96	1.2
PE(38:6) -H	762.51007	24.4	3.63E+05	4.28	2.31E+05	104.51	2.62E+05	76.24	1.0
PE(38:5) -H	764.52557	24.7	1.97E+05	4.39	2.27E+05	62.22	1.97E+05	33.29	1.2
PE(38:4) -H	766.54175	25.1	4.77E+04	4.79	3.30E+04	67.87	3.79E+04	62.20	0.9
PE(40:6p) -H	774.54633	25.6	7.94E+05	7.39	6.94E+05	92.42	7.11E+05	77.70	1.0
PE(40:6) -H	790.54132	25.2	1.52E+06	3.12	1.20E+06	91.45	1.13E+06	78.67	1.1

CV – coefficient of variation; LPE – lyso phosphatidylethanolamines; m/z – mass to charge ratio; PE – phosphatidylethanolamines; QC – quality control

Table SA3 Metabolites detected in RPLC-HRMS positive ion mode analysis and confirmed with fragmentation pattern.

Name + adduct	Observed m/z	Retention Time [min]	QC		White		Grey		WHITE/ GREY
			Mean	CV [%]	Mean	CV [%]	Mean	CV [%]	
Creatinine +H	114.0664	3.5	1.37E+05	11.66	2.16E+05	94.98	1.96E+05	39.94	1.1
Isothiocyanatobutane +H	116.0531	12.8	8.59E+03	7.95	8.70E+03	85.76	9.40E+03	118.97	0.9
norleucine +H	132.1019	7.3	9.81E+03	3.91	1.04E+04	75.21	1.61E+04	93.04	0.6
Deoxyribitol +H	137.0809	1.2	2.15E+04	9.37	6.48E+03	134.69	3.05E+03	47.03	2.1
Triethanolamine +H	150.1125	2.4	2.42E+05	4.63	6.40E+04	78.58	7.72E+04	130.01	0.8
Histidine +H	156.0768	2.0	1.79E+03	5.00	2.94E+03	87.33	8.36E+03	112.60	0.4
Phenylalanine +H	166.0865	1.4	2.24E+03	6.60	7.11E+02	36.32	8.84E+02	182.04	0.8
Artemisia alcohol acetate +H	197.1537	20.4	4.15E+05	1.80	1.18E+05	39.12	1.69E+05	39.78	0.7
Norlidocaine +H	207.1493	19.2	6.71E+05	1.37	1.17E+06	60.93	9.87E+05	44.43	1.2
tetradecenal +H	211.2058	18.7	1.69E+04	6.45	2.91E+04	56.05	2.53E+04	53.33	1.2
Furanofukinin+H	249.1848	23.4	3.70E+04	2.20	5.56E+04	68.36	6.18E+04	50.07	0.9
Hexadecenolide +H	253.2163	16.9	9.43E+05	16.32	1.06E+06	97.95	6.07E+05	42.10	1.7
Ginsenoyne D +H	263.2004	20.7	2.17E+05	3.03	2.75E+05	83.64	1.93E+05	133.56	1.4
Nandrolone +H	275.2005	22.7	4.23E+03	0.37	3.83E+03	54.48	3.40E+03	115.03	1.1
androstenol +H	275.2369	22.3	4.46E+05	7.33	2.20E+05	155.16	1.29E+06	101.06	0.2
Panaquinquecol 7 +H	277.1798	20.1	3.06E+05	12.81	2.13E+05	101.32	3.48E+05	147.85	0.6
Stearamide +H	284.2587	21.8	4.48E+03	0.37	2.44E+03	59.42	7.96E+03	105.35	0.3
Myristoylglycine +H	286.2373	20.4	6.09E+04	15.54	1.06E+05	78.84	9.94E+04	29.72	1.1
aminooctadecynediol +H	298.2739	20.7	1.87E+04	10.74	1.50E+04	55.50	1.68E+04	56.26	0.9
hydroxy stearic acid +H	301.2737	20.6	3.24E+04	8.68	3.54E+04	51.05	6.79E+04	41.75	0.5
Octadecatrienoic acid isobutylamide +H	334.3104	20.5	1.96E+04	16.53	7.77E+03	29.73	6.65E+03	15.61	1.2

Cholestadienone +H	383.3307	29.5	1.68E+04	4.20	9.82E+03	72.34	2.84E+04	44.75	0.3
dehydrocholesterol +H	385.3463	22.9	1.81E+04	14.21	5.38E+03	162.97	4.10E+04	87.79	0.1
Drotaverine +H	398.2327	22.3	2.44E+04	7.33	1.36E+04	49.38	3.94E+04	70.02	0.3
Hydroxymethyltetracosanoic acid +H	399.3832	24.8	3.78E+03	14.41	1.67E+03	109.35	9.18E+03	54.91	0.2
Squalene +H	411.3981	26.8	2.00E+04	16.55	2.21E+03	182.21	1.31E+04	140.87	0.2
Tasosartan +H	412.1874	26.8	2.28E+05	0.45	1.23E+05	54.57	2.94E+05	93.65	0.4
Diepomuricanin A +H	547.4719	26.9	8.20E+03	5.29	9.67E+03	72.37	1.44E+04	87.10	0.7
PE(38:6p) +H	748.5280	24.7	1.61E+05	4.04	2.09E+05	117.02	1.49E+05	86.40	1.4
PE(38:4p) +H	752.5590	25.5	1.55E+05	5.23	3.22E+05	78.39	2.30E+05	47.29	1.4
PE(38:6) +H	764.5227	24.3	2.46E+05	7.89	2.89E+05	111.46	2.12E+05	89.43	1.4
PE(38:4) +H	768.5536	25.1	3.10E+04	2.58	3.73E+04	101.81	4.72E+04	49.95	0.8
PE(40:6p) +H	776.5591	25.6	5.98E+05	1.90	8.41E+05	109.21	6.09E+05	98.47	1.4
PE(40:4p) +H	780.5897	26.4	1.24E+05	6.09	7.21E+04	150.72	1.40E+04	161.46	5.2
PE(40:6) +H	792.5539	25.2	1.07E+06	2.42	1.34E+06	114.17	1.02E+06	92.36	1.3
PE(40:5) +H	794.5694	25.8	5.34E+04	7.67	8.26E+04	69.98	6.67E+04	63.83	1.2
PE(40:4) +H	796.5852	26.0	1.89E+05	4.16	2.66E+05	111.96	1.94E+05	83.08	1.4

CV – coefficient of variation; m/z – mass to charge ratio; PE – phosphatidylethanolamines; QC – quality control

Table SA4 Lipid species detected in RPLC-HRMS analysis and confirmed with fragmentation pattern.

Name + adduct	Lipid Class	Observed m/z	Retention Time	Normalized peak area*						WHITE/ GREY	P-value
				QC		WHITE		GREY			
				Mean	CV	Mean	CV	Mean	CV		
LPC(16:0)+H	LPC	496.3397	5.4	0,001	8%	0,005	104%	0,003	117%	1,89	0,471
ChE(18:2)+NH_4	ChE	666.6183	15.3	0,006	1%	0,033	119%	0,014	144%	2,40	0,427
PE(34:1p)+H	PE	702.5432	11.7	0,007	4%	0,030	78%	0,008	130%	3,80	0,158
PE(36:2p)+H	PE	728.5565	11.8	0,014	2%	0,052	87%	0,015	141%	3,43	0,211
PE(36:1p)+H	PE	730.5745	12.3	0,005	6%	0,022	78%	0,006	137%	3,60	0,165
PE(38:4p)+H	PE	752.5589	11.8	0,004	27%	0,022	102%	0,005	113%	4,25	0,233
PE(38:4)+H	PE	768.5538	11.6	0,005	4%	0,022	128%	0,005	90%	4,35	0,318
PE(40:6p)+H	PE	776.5584	11.9	0,007	5%	0,028	99%	0,009	125%	3,21	0,266
PE(40:5p)+H	PE	778.5745	11.9	0,005	11%	0,020	72%	0,006	132%	3,36	0,154
PE(40:4p)+H	PE	780.5899	12.4	0,005	9%	0,022	74%	0,006	103%	3,80	0,140
PC(34:1)+Na	PC	782.5670	11.2	0,005	3%	0,023	102%	0,007	109%	3,48	0,262
PE(40:6)+H	PE	792.5536	11.6	0,009	5%	0,041	104%	0,012	114%	3,54	0,265
HexCer(d42:2)+H	HexCer	810.6814	12.5	0,010	1%	0,037	85%	0,011	142%	3,46	0,202
TG(48:2)+NH_4	TG	820.7386	14.8	0,014	1%	0,009	50%	0,017	59%	0,56	0,250
TG(48:1)+NH_4	TG	822.7541	15.0	0,010	1%	0,007	36%	0,008	45%	0,83	0,550
HexCer(d42:2+pO)+H	HexCer	826.6760	12.3	0,011	4%	0,041	76%	0,011	134%	3,53	0,162
TG(48:1)+Na	TG	827.7089	15.0	0,030	1%	0,020	69%	0,033	30%	0,62	0,203
HexCer(d42:1+O)+H	HexCer	828.6918	12.8	0,012	1%	0,043	94%	0,011	136%	3,99	0,212
TG(50:3)+NH_4	TG	846.7536	14.9	0,018	0%	0,010	52%	0,020	77%	0,47	0,269
TG(50:2)+NH_4	TG	848.7699	15.1	0,071	1%	0,037	58%	0,065	40%	0,56	0,144
TG(50:1)+NH_4	TG	850.7854	15.2	0,070	1%	0,046	69%	0,069	25%	0,66	0,253
TG(50:2)+Na	TG	853.7246	15.1	0,019	1%	0,012	26%	0,013	42%	0,86	0,581
TG(50:1)+Na	TG	855.7404	15.2	0,016	1%	0,012	34%	0,014	56%	0,86	0,677
TG(51:2)+NH_4	TG	862.7850	15.2	0,005	1%	0,004	61%	0,005	32%	0,81	0,555
TG(52:5)+NH_4	TG	870.7536	14.8	0,004	3%	0,002	57%	0,005	81%	0,51	0,324

TG(52:4)+NH_4	TG	872.7691	15.0	0,026	3%	0,015	64%	0,028	57%	0,52	0,209
TG(52:3)+NH_4	TG	874.7852	15.1	0,112	2%	0,064	70%	0,106	33%	0,60	0,194
TG(52:2)+NH_4	TG	876.8007	15.3	0,196	1%	0,124	73%	0,191	25%	0,65	0,259
TG(52:4)+Na	TG	877.7256	15.0	0,009	10%	0,006	44%	0,007	32%	0,76	0,338
TG(52:1)+NH_4	TG	878.8157	15.5	0,029	3%	0,024	98%	0,035	40%	0,69	0,468
TG(52:3)+Na	TG	879.7404	15.1	0,026	4%	0,018	45%	0,022	49%	0,84	0,612
TG(52:2)+Na	TG	881.7556	15.3	0,038	2%	0,028	42%	0,034	55%	0,82	0,601
TG(53:3)+NH_4	TG	888.8003	15.2	0,002	0%	0,002	79%	0,003	25%	0,67	0,321
TG(53:2)+NH_4	TG	890.8171	15.4	0,003	1%	0,003	89%	0,004	44%	0,74	0,542
TG(54:4)+NH_4	TG	900.8003	15.2	0,037	1%	0,020	63%	0,035	38%	0,57	0,152
TG(54:3)+NH_4	TG	902.8161	15.3	0,069	1%	0,039	67%	0,067	24%	0,58	0,131
TG(54:2)+NH_4	TG	904.8317	15.5	0,032	4%	0,023	85%	0,039	35%	0,59	0,225
TG(54:4)+Na	TG	905.7557	15.2	0,009	4%	0,006	42%	0,007	46%	0,86	0,641
TG(54:3)+Na	TG	907.7711	15.3	0,017	1%	0,011	42%	0,014	45%	0,76	0,420
TG(54:2)+Na	TG	909.7867	15.5	0,012	6%	0,008	54%	0,012	42%	0,67	0,290
TG(56:6)+NH_4	TG	924.8004	15.1	0,005	2%	0,003	46%	0,005	34%	0,57	0,097
TG(56:5)+NH_4	TG	926.8155	15.3	0,005	8%	0,003	57%	0,005	31%	0,62	0,159
TG(56:4)+NH_4	TG	928.8312	15.4	0,004	11%	0,002	82%	0,004	27%	0,56	0,160
TG(56:3)+NH_4	TG	930.8484	15.5	0,005	9%	0,003	84%	0,005	44%	0,53	0,181

*Ratio of the peak area and total peak area for lipids

ChE – cholesterol esters; CV – coefficient of variation; HexCer – hexosyl ceramides; LPC – lysophosphatidylcholines; m/z – mass to charge ratio; PC – phosphatidylcholines; PE – phosphatidylethanolamines; QC – quality control; TG – triglycerides

Table S.A.5. Lipid species detected in HILIC-HRMS analysis and confirmed with fragmentation pattern

Name + adduct	Lipid Class	Observed m/z	Retention Time [min]	Normalized peak area*						WHITE/ GREY	P-value
				QC		WHITE		GREY			
				Mean	CV	Mean	CV	Mean	CV		
LPC(16:1)+H	LPC	494.3241	8.9	0,001	17%	0,001	33%	0,001	56%	0,63	0,328
LPC(16:0)+H	LPC	496.3396	8.9	0,025	14%	0,031	18%	0,039	35%	0,80	0,484
LPC(18:2)+H	LPC	520.3398	8.5	0,001	25%	0,001	19%	0,002	48%	0,66	0,346
LPC(18:1)+H	LPC	522.3554	8.8	0,005	17%	0,007	19%	0,008	33%	0,87	0,741
LPC(18:0)+H	LPC	524.3711	8.7	0,007	14%	0,010	34%	0,010	25%	1,00	0,805
LPC(18:2)+Na	LPC	542.3217	8.8	0,001	5%	0,001	41%	0,001	55%	0,58	0,253
LPC(20:4)+H	LPC	544.3374	8.8	0,002	4%	0,002	28%	0,004	33%	0,70	0,226
LPC(20:3)+H	LPC	546.3530	8.7	0,002	3%	0,003	30%	0,003	30%	0,82	0,544
Cer(d36:1)+H	Cer	566.5507	1.3	0,001	3%	0,001	33%	0,001	11%	1,31	0,256
PE(34:2p)+H	PE	700.5271	7.6	0,002	10%	0,002	19%	0,003	22%	0,63	<0,05
PE(34:1p)+H	PE	702.5432	7.6	0,038	7%	0,033	32%	0,030	16%	1,08	0,867
PC(30:0)+H	PC	706.5381	6.8	0,014	14%	0,018	27%	0,013	12%	1,38	0,143
PC(32:1p)+H	PC	716.5589	7.5	0,003	11%	0,002	47%	0,002	36%	1,21	0,698
PC(31:1)+H	PC	718.5381	7.7	0,011	7%	0,012	24%	0,015	14%	0,81	0,335
PC(32:1e)+H	PC	718.5745	6.6	0,003	4%	0,004	28%	0,003	19%	1,15	0,439
PE(36:2p)+H	PE	728.5589	7.5	0,058	6%	0,056	64%	0,045	22%	1,25	0,668
PC(32:2)+H	PC	730.5381	6.6	0,001	6%	0,001	28%	0,001	16%	1,20	0,356
PC(32:1)+H	PC	732.5538	6.7	0,035	6%	0,038	19%	0,036	9%	1,06	0,616
PE(34:1)+Na	PE	740.5201	7.7	0,009	5%	0,007	39%	0,012	13%	0,60	<0,05
PC(33:2)+H	PC	744.5538	7.7	0,013	7%	0,013	5%	0,015	8%	0,86	0,058
PE(36:2p)+Na	PE	750.5408	7.4	0,044	12%	0,035	19%	0,041	11%	0,84	0,135
PE(38:5p)+H	PE	750.5415	7.4	0,047	11%	0,035	19%	0,041	14%	0,85	0,125
PE(36:1p)+Na	PE	752.5565	7.4	0,030	17%	0,033	46%	0,037	24%	0,89	0,751

PE(38:4p)+H	PE	752.5565	7.4	0,048	6%	0,047	20%	0,054	10%	0,88	0,355
PC(34:4)+H	PC	754.5357	6.6	0,005	21%	0,004	14%	0,005	7%	0,88	0,104
HexCer(d38:1)+H	HexCer	756.6348	3.1	0,001	11%	0,002	68%	0,001	41%	1,51	0,440
PE(36:2)+Na	PE	766.5376	7.5	0,043	7%	0,043	12%	0,047	5%	0,93	0,401
PE(36:1)+Na	PE	768.5514	7.5	0,002	8%	0,002	29%	0,002	21%	0,98	0,873
PC(36:4e)+H	PC	768.5878	6.6	0,005	6%	0,005	28%	0,005	23%	0,88	0,453
PE(38:4p)+Na	PE	774.5408	7.4	0,017	17%	0,016	29%	0,019	14%	0,88	0,476
PE(40:7p)+H	PE	774.5408	7.4	0,017	17%	0,016	29%	0,019	14%	0,88	0,489
PE(40:6p)+H	PE	776.5582	7.3	0,033	8%	0,038	27%	0,036	18%	1,05	0,673
PE(38:2p)+Na	PE	778.5745	7.4	0,033	9%	0,032	31%	0,030	18%	1,08	0,773
PC(34:2)+Na	PC	780.5514	6.6	0,011	8%	0,009	10%	0,015	19%	0,58	<0,05
PC(36:5)+H	PC	780.5538	6.6	0,011	8%	0,009	10%	0,015	19%	0,58	<0,05
PC(34:1)+Na	PC	782.5694	6.6	0,112	4%	0,101	19%	0,110	18%	0,91	0,460
PC(36:1)+H	PC	788.6157	6.6	0,099	16%	0,100	28%	0,073	10%	1,36	0,231
PE(38:4)+Na	PE	790.5357	7.5	0,011	8%	0,010	48%	0,013	37%	0,80	0,508
PE(38:3)+Na	PE	792.5531	7.5	0,044	13%	0,045	24%	0,046	28%	0,98	0,966
PC(36:2)+Na	PC	808.5827	6.5	0,022	13%	0,025	17%	0,024	8%	1,04	0,632
PC(36:1)+Na	PC	810.6007	6.5	0,043	10%	0,051	15%	0,040	9%	1,26	0,070
PC(38:4)+H	PC	810.6007	6.5	0,044	9%	0,055	29%	0,040	9%	1,36	0,176
HexCer(d43:2)+H	HexCer	824.6974	2.9	0,015	13%	0,015	55%	0,011	35%	1,32	0,549
PC(38:6)+Na	PC	828.5503	6.5	0,003	9%	0,003	26%	0,004	11%	0,69	<0,05
HexCer(d44:2)+H	HexCer	838.7123	2.8	0,010	13%	0,011	54%	0,007	28%	1,48	0,388
ST(d42:2)+H	ST	890.6376	1.7	0,005	2%	0,004	41%	0,004	27%	1,02	0,872

* Ratio of the peak area and total peak area for lipids

CV – coefficient of variation; HexCer – hexosyl ceramides; LPC – lysophosphatidylcholines; m/z – mass to charge ratio; PC – phosphatidylcholines; PE – phosphatidylethanolamines; QC – quality control; ST – sulfatides
